# Supplementary figures and images for: A Comprehensive 2D-LC/MS/MS Profile of the Normal Human Urinary Metabolome
Source: Diagnostics (Basel). 2022 Sep 9;12(9):2184. doi: 10.3390/diagnostics12092184 (PMC9497905; doi:10.3390/diagnostics12092184)

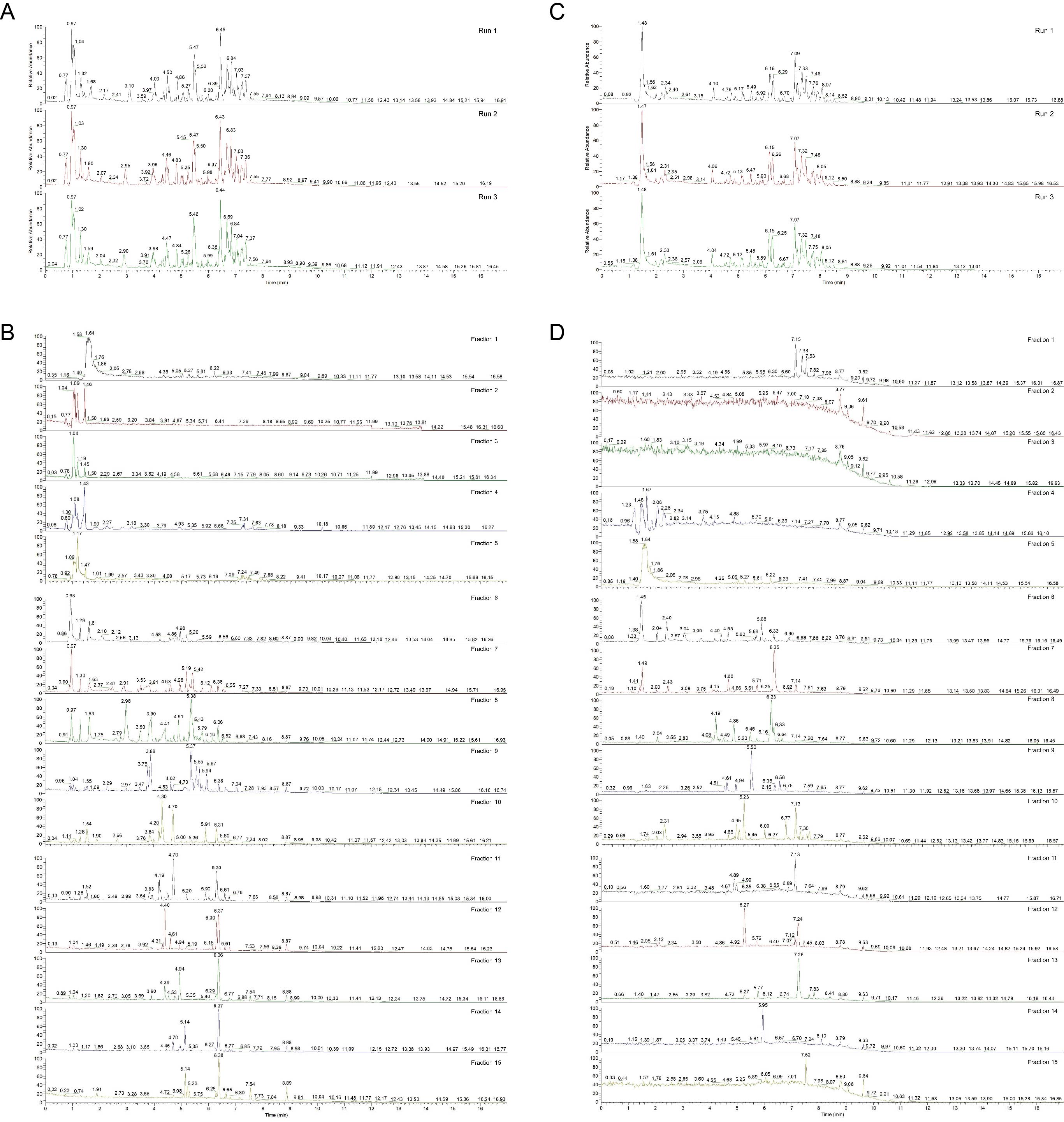

Supplement: Supplementary file 1 [file diagnostics-12-02184-s001.zip › Fig. S1.jpg]

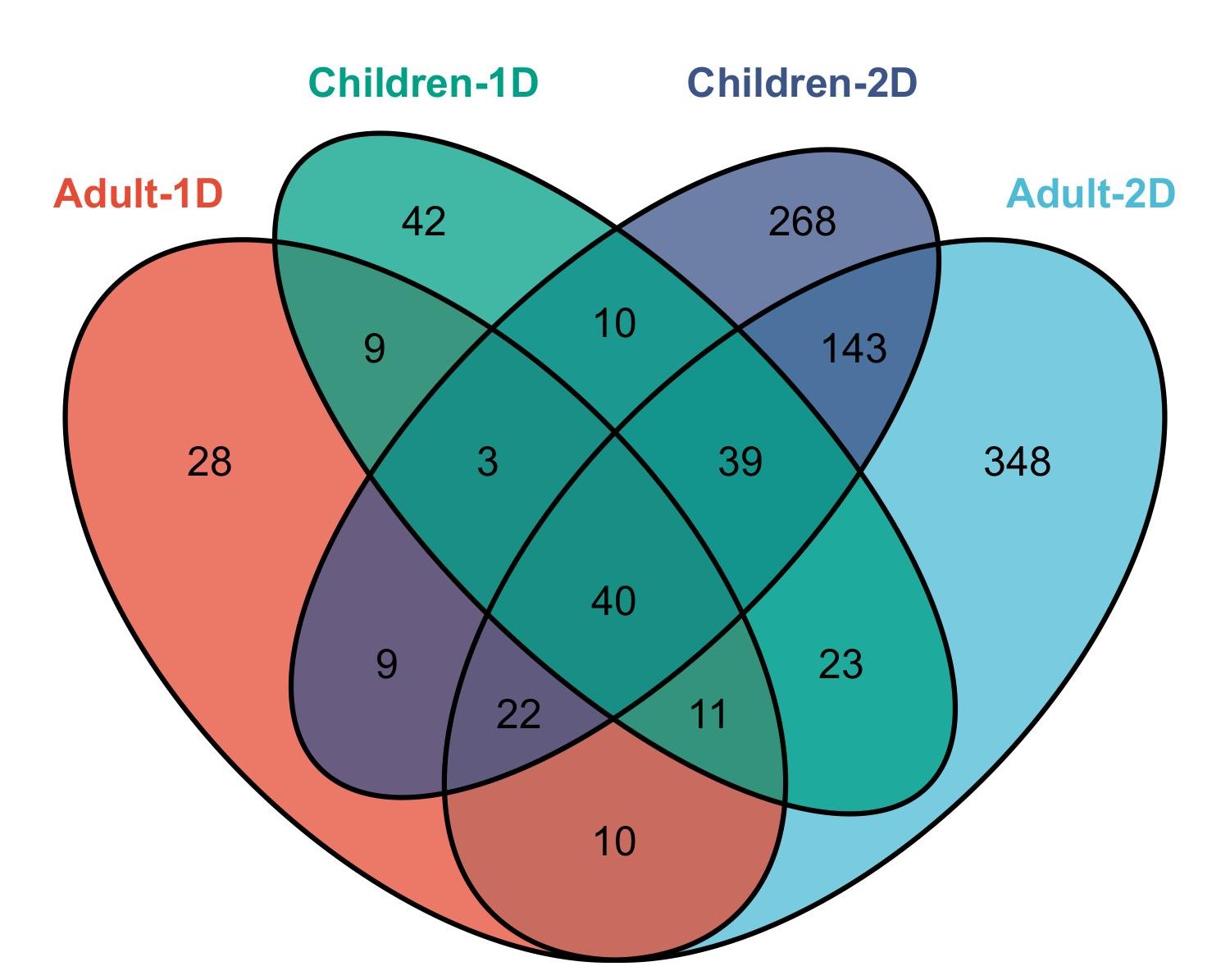

Supplement: Supplementary file 1 [file diagnostics-12-02184-s001.zip › Fig. S2.jpg]
